# High-intensity interval training in patients with glaucoma (HIT-GLAUCOMA): protocol for a multicenter randomized controlled exercise trial

**DOI:** 10.3389/fphys.2024.1349313

**Published:** 2024-05-16

**Authors:** Jan Van Eijgen, Valentin Schuhmann, Emma-Liina Fingerroos, Marie Renier, Holger Burchert, Julia Maria Kröpfl, Amaryllis Van Craenenbroeck, Véronique Cornelissen, Konstantin Gugleta, Ingeborg Stalmans, Henner Hanssen

**Affiliations:** ^1^ Department of Ophthalmology, University Hospitals UZ Leuven, Leuven, Belgium; ^2^ Research Group Ophthalmology, Department of Neurosciences, Leuven, Belgium; ^3^ Department of Sports, Exercise and Health, Medical Faculty, University of Basel, Basel, Switzerland; ^4^ Research Group of Rehabilitation of Internal Disorders, Department of Rehabilitation Sciences, Faculty of Movement and Rehabilitation Sciences, Leuven, Belgium; ^5^ Division of Nephrology, University Hospitals UZ Leuven, Leuven, Belgium; ^6^ Nephrology and Renal Transplantation Research Group, Department of Microbiology, Immunology, and Transplantation, Leuven, Belgium; ^7^ Department of Ophthalmology, University of Basel, Basel, Switzerland

**Keywords:** glaucoma, retinal microcirculation, exercise, high intensity interval training, cardiovascular risk, intraocular pressure

## Abstract

**Background:**

Glaucoma stands as a prominent global cause of irreversible blindness and the primary treatment approach involves reducing intraocular pressure (IOP). However, around one-third of patients exhibit disease progression despite effective IOP reduction. Microvascular endothelial function, chronic inflammation, and oxidative stress are known to affect retinal neuronal networks and have been associated with disease severity and progression. Exercise training has the potential to counteract these mechanisms as add-on treatment to usual care.

**Aims:**

The HIT-GLAUCOMA study will investigate the effects of a 6-month high-intensity interval training (HIIT) on intermediate endpoints such as local retinal microvascular and systemic large artery function, inflammation, and oxidative stress as well as clinical endpoints such as visual field indices, optic nerve rim assessment, retinal nerve fiber layer thickness, IOP, number of eye drops, vision-related quality of life and ocular surface disease symptomatology.

**Methods:**

The study is a multi-center randomized controlled clinical trial in patients with both normal tension and high-tension primary open angle glaucoma. Across two study centers, 128 patients will be enrolled and randomized on a 1:1 basis into an exercise intervention group and a usual care control group. The primary microvascular endpoints are retinal arteriolar and venular flicker light-induced dilation at 6 months. The primary endpoint in the systemic circulation is brachial artery flow-mediated dilation at 6 months.

**Anticipated results:**

We hypothesize that exercise therapy will improve retinal microvascular function and thus ocular blood flow in patients with glaucoma. As clinical outcomes, we will investigate the effect of exercise on visual field indices, optic nerve rim assessment, retinal nerve fiber layer thickness, IOP, number of eye drops, vision-related quality of life and ocular surface disease symptomatology.

**Discussion:**

HIT-GLAUCOMA is a blueprint trial design to study the effect of exercise training on neurodegenerative and cardiovascular diseases. Importantly, patients are also expected to benefit from improvements in general health and cardiovascular co-morbidities. If proven effective, exercise may offer a new add-on treatment strategy to slow glaucoma progression.

**Clinical Trial Registration Number:**

The trial is registered at Clinicaltrials.gov under the identifier NCT06058598 and is currently in the recruitment stage.

## Introduction

### Glaucoma and cardiovascular risk

Glaucoma is an optic neuropathy characterized by degeneration of retinal ganglion cells, thinning of the retinal nerve fiber layers, and cupping of the optic disc (Jonas et al., 2017). Primary open angle glaucoma (POAG) can be sub-classified according to the maximal intraocular pressure (IOP). This arbitrary classification with a cut-off at 21 mmHg IOP stratifies POAG patients into high-tension glaucoma (HTG) and normal tension glaucoma (NTG) (BMJ Publishing Group Ltd, 2021). It is hypothesized that impaired ocular perfusion pressure, impaired microvascular endothelial function and increased oxidative stress are additional disease drivers (Chu-Tan et al., 2022; Benoist d Azy et al., 2016; Melgarejo et al., 2023). The glaucomatous changes lead to a loss of visual field (Venkataraman et al., 2010; Quigley, 2011), and in around 15%–20% of glaucoma cases the disease progresses to blindness (Susanna et al., 2015). Glaucoma has become the global leading cause of irreversible blindness (Topouzis et al., 2007). It affects over 80 million people worldwide (Topouzis et al., 2007) and the prevalence is estimated to increase to 111 million in 2040, in accordance to population ageing (Tham et al., 2014). The main goal of glaucoma treatment is to lower IOP. However, at least one-third of patients show disease progression despite IOP lowering (Peters et al., 2014; Susanna et al., 2015).

Some subclinical pathophysiological features of cardiovascular diseases (CVD) overlap with glaucoma, suggesting an association between CVD and glaucoma (Choi et al., 2023). A growing body of evidence indicates that glaucoma is associated with many systemic CV risk factors such as obesity, hypertension, active smoking status, low high-density-lipoprotein (HDL), high low-density-lipoprotein (LDL) levels and hypertriglyceridemia (Lin et al., 2010; Kreft et al., 2019; Talaat et al., 2021; Wändell et al., 2022). Primary and secondary vascular dysregulations have previously been hypothesized as components of the etiology of glaucoma (Flammer and Konieczka, 2017). Earlier research demonstrated that systemic comorbidities associated with endothelial lesions, atherosclerosis and hypoperfusion may lead to damage of the retinal nerve fiber layer and the underlying connective tissue (Dascalu et al., 2021). Several studies have shown both hypertension, nocturnal hypotension and daytime blood pressure variability to be common CV risk factors for progression (Dascalu et al., 2021; Van Eijgen et al., 2023). Glaucoma may therefore be considered as an independent risk predictor of new-onset CVD (Choi et al., 2023), and *vice versa*. Conversely, glaucoma patients with healthy lifestyle behaviors have shown a reduced risk for incident CVD compared to those with unhealthy behaviors (Choi et al., 2023). Thus, the importance of vascular and other non-IOP related risk factors underscore the need for screening of alternative biomarkers of glaucoma and by extension CV comorbidities.

To date, research on the association of glaucoma disease with large artery structure and function in the systemic circulation is scarce. Since patients with glaucoma suffer from CV co-morbidities and present with systemic inflammation and oxidative stress, they are prone to develop large artery endothelial dysfunction and unfavorable arterial stiffness. A holistic approach including the assessment of brachial artery flow-mediated dilation (FMD) and pulse wave velocity (PWV), both being large artery surrogate markers for CV risk (Vlachopoulos et al., 2010; Thijssen et al., 2011), may provide a more thorough understanding of the bidirectional relation between systemic vascular disease and local neurodegenerative eye disease.

### Glaucoma and the retinal microcirculation

The blood flow in the retina maintains a constant perfusion level through the intricate interplay between neurovascular coupling, myogenic responses, flow-mediated mechanisms, and metabolic processes, facilitated by the release of vasoactive substances from the endothelium and retinal tissue (Koller and Toth, 2012). The capillary endothelial cells and the vascular smooth muscle cells are responsible for the pressure-dependent myogenic constriction (Bayliss-effect) and flow-dependent nitric oxide (NO)-induced dilation by means of shear stress (Hanssen et al., 2022). In addition, endothelin-1 (ET-1) has been suggested to play an important role in the maintenance and regulation of local blood flow (Palmer et al., 1988; Moncada and Higgs, 1993). Together with NO, ET-1 ensures a balance between vasoconstrictive and vasodilatory capacities of the endothelium (Koller and Toth, 2012).

Local oxidative stress has been acknowledged as a factor in POAG pathogenesis for over 30 years. Since then, several studies have supported the role of free radical-induced oxidative stress in trabecular meshwork (TM) cell loss (Zhou et al., 1999). The retina is consistently exposed to reactive oxygen species (ROS) (Nita and Grzybowski, 2016). An imbalance between ROS production and antioxidant defenses can lead to cellular damage and apoptosis (Chrysostomou et al., 2013; Pinazo-Durán et al., 2015). Elevated IOP and visual field damage have been linked to oxidative DNA damage affecting TM cells (Saccà et al., 2005; Nita and Grzybowski, 2016). In addition, neurotrophic factors (NTFs), in particular brain-derived neurotrophic factor (BDNF), have been identified in the pathogenesis of glaucoma (Lambuk et al., 2022). BDNF is locally regulated in the retina and transported from the brain to the retina through retrograde transport (Lambuk et al., 2022). Disruptions in the retrograde transport and reduced levels of BDNF in the serum and ocular fluids of patients with glaucoma suggest that neurotrophic deprivation contributes to glaucomatous optic neuropathy (Lambuk et al., 2022).

Retinal microvascular endothelial dysfunction is a key predictive vascular parameter, along with arterial blood pressure and vascular rarefaction (Gugleta et al., 2012; Wang et al., 2015; Kwon et al., 2017). Associations of retinal vessel diameters and function with CV risk and disease have previously been extensively reviewed (Hanssen et al., 2022). Narrowing of the central retinal arteriolar diameter equivalent (CRAE), widening of central retinal venular diameter equivalent (CRVE), and a lowering of arteriolar-to-venular diameter ratio (AVR) associate with an increased risk of CVD and CV mortality (Green and Smith, 2018; Vera et al., 2019). A similar retinal vessel diameter constellation was found in POAG patients when compared to healthy subjects (Chiquet et al., 2020). Moreover, impaired retinal flicker-induced dilation (FID) correlates with increased CV risk in diabetes (Valenzuela et al., 2023), hyperglycemia (Green and Smith, 2018), hypertension (Vera et al., 2019), dyslipidemia (Karakucuk et al., 2020), obesity (Kaminsky et al., 2013), coronary artery disease (Ramos et al., 2015), and chronic kidney disease (Whyte et al., 2013). Blood flow alterations in the optic nerve head (ONH) have been identified as a significant risk for development and progression of glaucoma, highlighting the importance of the vascular system’s responsiveness to metabolic changes for disease progression (Flammer et al., 2002).

### Exercise treatment in patients with glaucoma

Regular physical activity and exercise could potentially reduce the main underlying mechanisms in the development and progression of CVD, such as inflammation, oxidative stress and endothelial dysfunction (Green et al., 2017; Valenzuela et al., 2023). The effects of exercise training are mediated through a repeated hemodynamic stimulation occurring during each bout of exercise. This stimulation leads to an increase in the production and bioavailability of endothelium-derived substances such as NO. Regular bouts of exercise can induce functional and structural vascular adaptations that improve systemic and local blood flow as well as oxygen supply (Green and Smith, 2018). Additionally, HIIT temporarily reduces IOP values in physically active young subjects (Vera et al., 2019; Karakucuk et al., 2020).

Cardiorespiratory fitness inversely correlates with CV mortality and morbidity (Kaminsky et al., 2013). High-intensity interval training (HIIT) appears to be the most promising candidate to increase this fitness and vascular health (Wisløff et al., 2009; Whyte et al., 2013; Ramos et al., 2015). Furthermore, emerging evidence indicates that HIIT effectively reduces oxidative stress and increases the bioavailability of NO (Areas et al., 2019). When compared to low-to-moderate intensities, exercising in intervals and at higher intensities has been shown to be superior in improving insulin sensitivity, glucose metabolism, HDL and LDL, left ventricular dysfunction, NO bioavailability, endothelial function, as well as cardiorespiratory fitness (Tjønna et al., 2008; Wisløff et al., 2009; Whyte et al., 2013; Weston et al., 2014; Ramos et al., 2015; Streese et al., 2018). Substantial evidence suggests that not only local, but also systemic vascular abnormalities play an important role in the pathophysiology of glaucoma (Bonomi et al., 2000; Dascalu et al., 2021). In glaucoma patients, dynamic exercise such as jogging and cycling has been shown to lower IOP, with longer and more intense training having greater effects (Streese et al., 2018). Exercise also demonstrated to induce higher levels of BDNF and improve mitochondrial function (Zhu et al., 2018; Chu-Tan et al., 2022). Notably, having patients with glaucoma adopting an active lifestyle is beneficiary for their general health, and concomitant management of CV risk factors is an effective means to improve CV health and quality of life.

## Methods and analysis

The long-term clinical goal of HIT-GLAUCOMA is to slow glaucoma progression through an exercise intervention. This trial will investigate the effects of HIIT on retinal microvascular structure and function, systemic large artery health as well as systemic inflammation and oxidative stress in patients with glaucoma. These mechanisms represent the intermediate endpoints and are known to influence glaucoma progression.

### Objectives and outcome measures

#### Primary

• Retinal microvasculature (local): the primary objective in the retinal microcirculation is to examine the effects of a 6-month HIIT-intervention on maximal flicker light-induced retinal arteriolar (aFID) and venular (vFID) dilation as a marker of microvascular endothelial function in patients with glaucoma. We hypothesize that exercise treatment significantly increases retinal aFID and vFID when compared to the control group.

Large arteries (systemic): the primary objective in the macrocirculation is to examine the effects of a 6-month HIIT-intervention on FMD as a marker of large artery endothelial function in patients with glaucoma. We hypothesize that exercise treatment significantly increases FMD as compared to the control group.

#### Secondary

• To investigate whether 6 months of exercise training improve CRAE and CRVE diameters in glaucoma patients compared to the control group.
• To investigate whether 6 months of exercise training improves PWV as a marker of large artery stiffness in glaucoma patients compared to the control group.
• To investigate retinal aFID and vFID, other flicker light derived markers, as well as retinal CRAE and CRVE after 6 months of exercise training and a further 6 months of self-maintenance exercise training as compared to the control group at 12 months.
• To investigate large artery FMD and PWV after 6 months of exercise training and a further 6 months of self-maintenance exercise training as compared to the control group at 12 months.
• To investigate whether 6 months of exercise training increase brain-derived neurotrophic factor (BDNF) levels and reduce inflammation as well as oxidative stress in peripheral blood. To assess IOP, use of IOP lowering eye drops and occurrence of laser trabeculoplasty or surgical interventions after six and 12 months in the intervention group as compared to the control group.
• To assess Optical Coherence Tomography (OCT) and visual field metrics at six and 12 months in the intervention group as compared to the control group.
• To assess peak aerobic capacity (VO_2_peak) and objectively measured physical activity at six and 12 months in the intervention group as compared to the control group.
• To compare changes in 24 h ambulatory blood pressure measurement and blood pressure variability at six and 12 months in the intervention group and the control group.
• To compare changes in vision-related quality of life at six and 12 months in the intervention group and the control group.
• To compare changes in questionnaire-derived ocular surface disease index and dry eye disease symptoms in the intervention group and the control group.

### Study design

The HIT-GLAUCOMA study is a multi-center, prospective, randomized controlled, clinical intervention study. Two centers are involved: The University of Basel (Switzerland) comprised of the Department of Sport, Exercise and Health (DSBG) and the Department of Ophthalmology, and the University of Leuven (KU Leuven, Belgium), comprised of the Department of Ophthalmology, the Department of Neurosciences and the Department of Rehabilitation Sciences. In Basel, the clinical ophthalmological examination will be carried out during the glaucoma consultation at the University Eye Clinic, while retinal vessel analyses, systemic vascular diagnostics and further analysis including cardiopulmonary exercise testing (CPET) and exercise training will be performed at the DSBG. In Leuven, clinical ophthalmological assessments, retinal vessel analyses and further analyses will be performed during the glaucoma consultation at the University Eye Clinic (UZ Leuven, Belgium), while systemic vascular diagnostics and CPET as well as exercise training will be performed at the Department of Rehabilitation Sciences.

**FIGURE 1:**
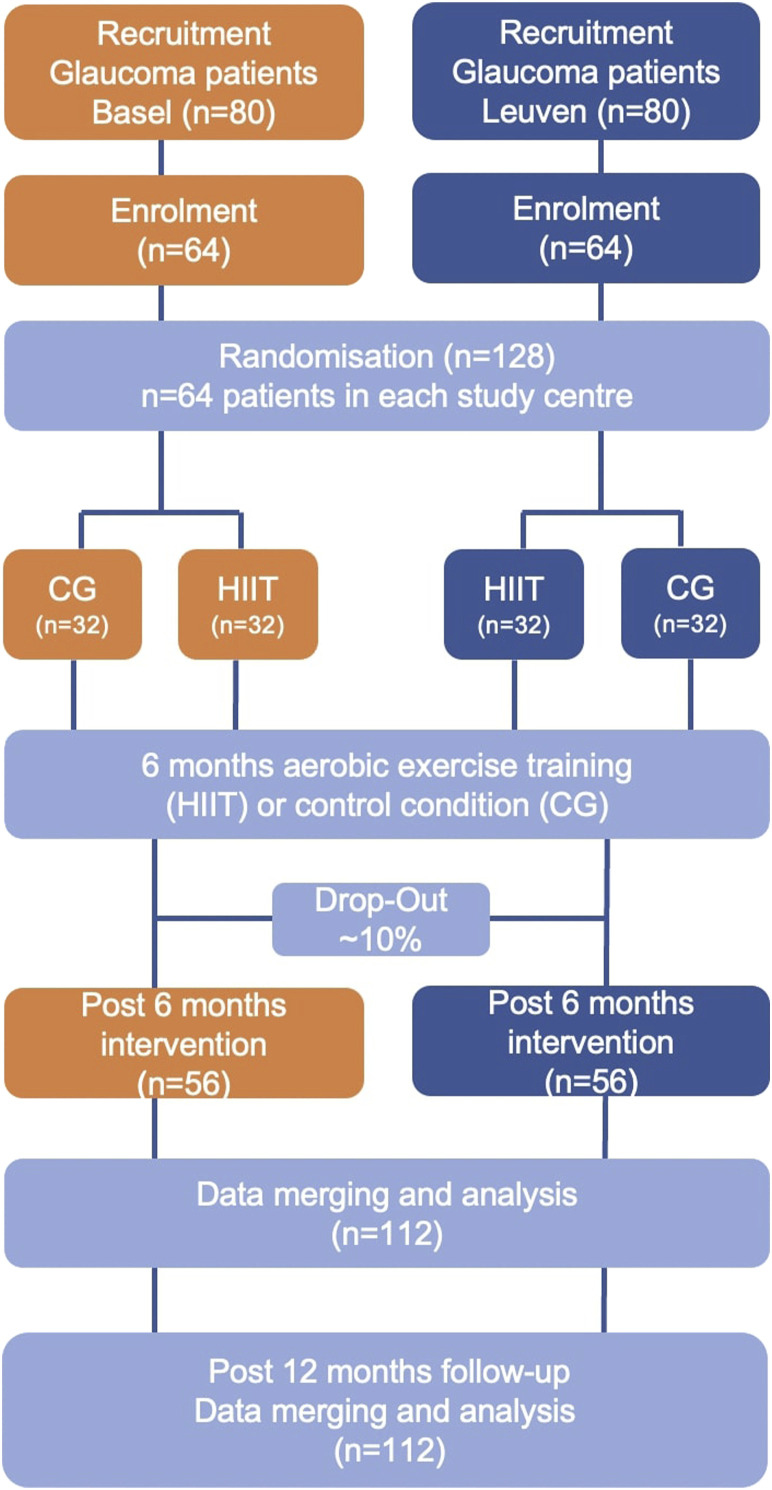
Trial flow chart.

In the HIT-GLAUCOMA study, the effects of a 6-month exercise intervention in patients with glaucoma (*n* = 64) are compared to a control group of glaucoma patients (*n* = 64) who receive guideline-based physical activity recommendations (150–300 min of moderate-intensity aerobic physical activity, or 75–150 min of vigorous-intensity activity, or equivalent combination of both throughout the week), while accounting for concomitant changes in glaucoma standard-of-care medication. Patients with NTG and HTG will be randomized into two groups (Figure 1): the intervention group (HIIT; *n* = 32 per center) will take part in a combined and progressive moderate continuous training (MCT) and HIIT; the control group (CG; *n* = 32 per center) will receive physical activity recommendations as control condition. Exercise training is offered as adjunct treatment to the standard of care. The supervised exercise intervention will terminate after 6 months followed by 6 months of unsupervised self-maintenance exercise training or control condition.

### Selection of subjects

#### Inclusion criteria

Patients eligible for inclusion must meet all of the following criteria: age 40–80 years; diagnosed with POAG (both NTG and HTG); being in regular follow-up in either of the two study centers and having signed written informed consent.

#### Exclusion criteria

Patients eligible for this study must not meet any of the following criteria: having had glaucoma surgery within 6 months before start of the project; changes in topical medication or laser trabeculoplasty within 3 months before start of the project; severe glaucoma (visual field mean deviation lower than −12Db); eye disease that has a significant impact on the visual field; life-threatening arrhythmia, aberrant CPET (e.g., signs of ischemia) which precludes participation in an exercise trial; insulin-treated diabetes, chronic obstructive pulmonary disease or cancer; mental, clinical or physical limitation precluding safe participation in a high intensity exercise program. Subjects that perform more than 150 min of moderate to vigorous physical activity per week as defined by the Physical Activity Vital Sign questionnaire are also excluded (Kuntz et al., 2021). All participants considered for study participation, per the above criteria, will be documented on the Screening Log, including screening failures.

#### Recruitment and randomization

Eligible glaucoma patients will invited to participate during their routine glaucoma consultation at the Eye Clinic of the University Hospital Basel or the Glaucoma Clinic of the University Hospitals UZ Leuven. Overall, 128 patients will be enrolled: 64 POAG (HTG and NTG) patients in Basel and 64 in Leuven.

Participants will be randomized to either HIIT or CG, stratified for the study site. Randomization is carried out via Redcap^®^ to ensure concealed allocation and minimize selection bias. Block randomization will be performed (blocks 4–6) via Redcap^®^. Patients will receive a unique identification number, which will be used on all subsequent transcripts, on all collected data and in the Redcap^®^ database instead of the participant´s name to assure pseudonymity of all data on all documents and in all web-based platforms. The following randomization procedures have been established: single blinding of the study personnel involved in the assessments during study visits to avoid motivational bias. Analysis of imaging data will be performed off-line by blinded researchers.

## Interventional methods

### Intervention group

**FIGURE 2:**
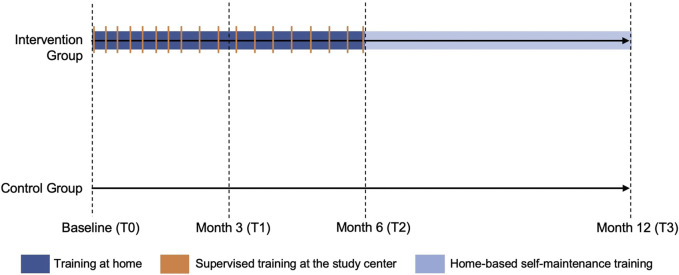
Overview of the training sessions in the exercise intervention and control group.

Patients randomized to the exercise intervention group will follow a 6-month long progressive exercise training program. The program consists out of 6 weeks of MCT, followed by 20 weeks of MCT combined with HIIT. This 6-month period is followed by a maintenance, non-supervised, home-based physical activity program (Figure 2). During the first 6 months patients will participate in 18 supervised center-based exercise sessions in adjunct to a remotely monitored and guided home-based exercise intervention. The number of supervised center-based sessions will gradually decrease over time to encourage self-management and empowerment: during the first and second month, patients will participate in one supervised training session per week. Starting from the third month until the sixth, patients will participate in one supervised session every second week.

The 6-month exercise program consists of three phases. Aerobic training is performed three times per week on a cycle ergometer. Phase one is a 6-week run-in period to acquaint patients with the exercise therapy and allow a gradual and progressive aerobic conditioning. During this phase, three sessions of MCT are performed. In phase two, one session of MCT will be replaced by a HIIT session. During phase three, patients will execute two sessions of HIIT and one session of MCT. Duration and intensity of the training sessions are specified within Table 1. Intensity of the training sessions is guided by heart rate monitoring, corresponding to a specific percentage of the VO_2_ reserve. VO_2_ reserve is defined as ((VO_2_max—VO_2_rest) x %)—VO_2_rest) (Swain and Leutholtz, 1997). Patients will monitor their exercise (intensity, duration, frequency) by means of a commercial wearable exercise tracker and will be asked to upload their training data to the accompanying platform. In addition, their self-reported rate of perceived exertion is collected in an individualized training log. The 6-month exercise intervention will be followed by a maintenance physical activity program tailored to the patients’ preferences during a 60-min face-to-face consultation. The last 6 months of exercise are not supervised nor further supported by the study team.

**TABLE 1:** Exercise intervention.

	Phase 1 Week 1–6	Phase 2 Week 7–12	Phase 3 Week 13–26
Supervised session	Week 1–6: 1x/week	Week 7–10: 1x/week Week 11–12: 1x/2 weeks	Week 13–26: 1x/2 weeks
Modality/week	3 x MCT	2 x MCT +1 x HIIT	1 x MCT +2 x HIIT
Warm-up	5 min, ramp to 25% VO_2_reserve	5 min, ramp to 25% VO_2_reserve	5 min, ramp to 25% VO_2_reserve
Duration (conditioning phase)	Week 1: 30 min	MCT: 45 min HIIT: 30 min [(6 × 2 min)+(6 × 3 min)]	MCT: 45 min HIIT: 28 min [(4 × 4 min)+(4 × 3 min)]
	Week 2: 35 min		
	Week 3: 35 min		
	Week 4: 35 min		
	Week 5: 40 min		
	Week 6: 45 min		
Intensity (conditioning phase)	Week 1–2: 50% VO_2_reserve Week 3–6: 65% VO_2_reserve	MCT	MCT
		65% VO_2_reserve	65% VO_2_reserve
		HIIT	HIIT
		6 × 2 min	4 × 4 min
		1 min ramp until 80% VO_2_reserve followed by 1 min sustaining 80% VO_2_reserve	1 min ramp until 85% VO_2_reserve followed by 3 min sustaining 85% VO_2_reserve
		Interspersed by 6 × 3 min	Interspersed by 4 × 3 min
		50% VO_2_reserve	60% VO_2_reserve
Cool down	5 min	5 min	5 min

### Control group

The CG will verbally receive an exercise recommendation that is in line with current guidelines (Pelliccia et al., 2020). We will recommend physical activity of at least 150 min per week at a moderate to high intensity. No counselling or guidance on objective measures of physical activity by means of wearables will be provided.

### Concomitant care

Both intervention and control group will continue to receive all routine medical and pharmacological management.

### Assessments

An overview of all methods applied and a timetable of the scheduled visits is given in Table 2. The following section offers a summary of the diagnostic methods.

**TABLE 2:** Examination overview for each visit.

Overview of examinations*	T0	T1	T2	T3	T4
Glaucoma Standard of Care					
Visual Acuity	X		X	X	X
Autorefraction/autokeratometry	X		X	X	X
Visual Field	X		X	X	X
Intraocular Pressure	X		X	X	X
Retinal Photography	X		X	X	X
Optical Coherence Tomography	X		X	X	X
Retinal Microvascular Assessments					
Static Retinal Vessel Analysis	X	X	X	X	
Dynamic Retinal Vessel Analysis	X	X	X	X	
Laser Speckle Flowgraphy (Basel)	X	X	X	X	
Optical Coherence Tomography Angiography	X	X	X	X	
Large Artery Structure and Function					
Flow Mediated Dilation	X	X	X	X	
Pulse Wave Velocity	X	X	X	X	
Physical Activity and Fitness					
Cardiopulmonary Exercise Testing (VO_2_peak)	X	X	X	X	
Accelerometer	X	X	X	X	
Further Assessments					
Anthropometry (Weight, Height, BMI, BP, ECG)	X	X	X	X	
24 h Ambulatory Blood Pressure Measurement	X	X	X	X	
Blood and Urine Sampling	X	X	X	X	
*Safety Monitoring*	*X*	*X*	*X*	*X*	
Questionnaires					
International Physical Activity Questionnaire (iPAQ)	X	X	X	X	
National Eye Institute 25-Item Visual Function Questionnaire (NEI VFQ-25)	X	X	X	X	
Glaucoma Activity Limitation questionnaire (GAL-9)	X	X	X	X	
Ocular Surface Disease Index (OSDI)	X	X	X	X	
Five-item Dry Eye Questionnaire (DEQ-5)	X	X	X	X	

T0, baseline; T1, measurement at 3 months; T2, measurement at 6 months; T3, measurement at 1 year; T4, yearly until 16 years; BMI, body mass index; BP, blood pressure; ECG, electrocardiography. *Safety will be monitored throughout the whole trial.

#### Clinical ophthalmological assessments

Visual field examination: Visual Field quantification is the gold standard for assessment of visual performance in glaucoma patients and are quintessential for glaucoma diagnosis and follow-up. The Humphrey visual field (Carl Zeiss Meditec, Inc., Dublin, CA) and Octopus visual field (Haag-Streit AG, Koeniz, Switzerland) are used for the examination.

Optical coherence tomography (OCT): OCT makes use of interferometry in the visible wavelength spectrum to make orthogonal slices of the retina in high resolution. Scanning of the macular retinal thickness (ganglion cell layer), retinal nerve fiber layer thickness and optic nerve rim assessment will be performed using Heidelberg Spectralis (Heidelberg Engineering Inc., Franklin, United States). In particular, a macular OCT cube as well as radial and circumpapillary scans across the ONH will be taken.

Optical coherence tomography angiography (OCTA): OCTA imaging can be best compared to subtraction angiography. It subtracts OCT images from each other, in which the only mobile part is the moving blood column. The result is a three-dimensional reconstruction of the retinal vascular tree to the level of the smallest capillaries. 10° and 20° vascular scans of the macula and 15° scans of the optic nerve will be captured using Heidelberg Spectralis (Heidelberg Engineering Inc., Franklin, United States).

Visual acuity testing: Visual acuity will be measured using a center-specific calibrated visual acuity chart and expressed in the universal Logarithm of the Minimum Angle of Resolution (LogMar) scoring.

Autorefraction/autokeratometry: The refraction (sphere, cylinder and axis) will be measured using an automated keratorefractometer. The optical qualities (refraction) of the eye are important for visual acuity testing and optical adjustment of imaging.

Intraocular pressure (IOP): IOP is measured by use of Goldmann Applanation Tonometry prior to pupil dilation.

#### Retinal microvascular assessments

Dynamic retinal vessel analysis: aFID and vFID are assessed as biomarkers of endothelial function of the retinal microcirculation. The dilation in response to flicker light is facilitated by neurovascular coupling. Dilation capacity in percent change from the baseline diameter will be assessed with the Dynamic Vessel Analyzer 3.0 (DVA, IMEDOS Systems, Jena, Germany) (Hanssen et al., 2022). The device consists of a retinal camera (Imedos Systems, Jena, Germany), a charge-coupled device camera for electronic online imaging and a personal computer for system control, analysis, and recording of the obtained data. Three cycles of flicker provocation are applied with 80 s of recovery after each of the cycle to extract a.o. the mean value for aFID and vFID (Streese et al., 2021).

Static retinal vessel analysis: Taking a fundus image of the retina allows for the assessment of CRAE and CRVE diameters as well as AVR. These parameters have been shown to reflect systemic CV risk when analyzed in the healthy eye and represent retinal microvascular health (Hanssen et al., 2022). Retinal vessel images will be taken using the Static Retinal Vessel Analyzer (SVA-T, Imedos Systems UG, Jena, Germany). The SVA-T system consists out of a fundus camera (Topcon TRC NW8) and analyzing software (Visualis 2.80, Imedos System UG). Three images will be taken from the retina of the affected eye. Retinal vessel diameters will be identified and analyzed by use of a semi-automated software (Vesselmap 2, Visualis, Imedos Systems UG) and automated softwares (LUNet and PVBM) (Fhima et al., 2023).

Laser speckle flowgraphy (LSFG): The ocular perfusion will be measured with laser flowgraphy (mean blur rate, MBR, as estimate for flow) with LSFG (Nidek Inc., United States) only at the Basel site. The device uses a low-power semi-conductor laser (wavelength of 830 nm, laser class 1) for its measurement light. LSFG measures real-time fundus blood flow. The relative blood flow is expressed as an arbitrary unit of MBR. A total of 118 frames will be recorded for 4 s. Mean perfusion in the ONH will be analyzed with LSFG Analyzer software (version: 1.0.0.0).

#### Macrovascular assessments

Flow-mediated dilation: FMD is the gold standard method for non-invasive evaluation of endothelial function in large arteries. Using an ultrasound-based device (2D imaging), dilation of the brachial artery is measured for 3 minutes after 5 minutes of cuff-induced blood flow restriction (UNEX Sakae Nakaku Nagoya; Japan). The diameter of the brachial artery is measured at rest (rest diameter), shortly before cuff deflation (base diameter) and during 3 minutes of increased blood flow (max diameter) to analyze flow and endothelium-dependent vasodilation. Blunted FMD has been linked to higher CV event risk and it has been shown to have a prognostic value for development of CVD (Ras et al., 2013).

Pulse-wave velocity and analysis: Carotid-femoral pulse wave velocity and estimates of the central aortic blood pressure will be measured using the SphygmoCor XCEL (Atcor Medical, 184 Shuman Blvd, Naperville, United States). PWV is the gold standard for the measurement of arterial stiffness (Chalmers et al., 2010). A good intra- and interobserver reproducibility of this technique has been demonstrated in healthy populations and in patients with elevated CV risk (Streese et al., 2020b). After 10 minutes of rest, pulse waves are recorded using a high-fidelity tonometric transducer at the carotid site with simultaneous recording of pulse waves using a cuff-based oscillometric detection at the upper leg. Pulse wave distance is determined by use of the direct method, as recommended in a previous consensus document (Van Bortel et al., 2012). The distance from the carotid artery to the top of the cuff is measured, followed by the distance from the femoral artery to the top of the cuff. Distance between the carotid and femoral palpation site is calculated via subtraction. The measurements meet quality control parameters if three consecutive measurements are visually acceptable and within 0.5 m/s of each other with a standard deviation of <10%. The mean of all valid measurements represents the PWV value.

#### Exercise capacity testing

Cardiopulmonary exercise testing (CPET): Exercise capacity will be assessed by CPET to measure peak exercise oxygen consumption (VO_2_peak). CPET is performed on an electromagnetically braked cycle ergometer (Ergoline 900, Bitz, Germany) according to the standards of the American Thoracic Society/American College of Chest Physicians’ statement on cardiopulmonary exercise testing (American Thoracic SocietyAmerican College of Chest Physicians, 2003). Individualized ramp protocol will be performed until volitional exhaustion or appearance of clinically significant symptoms. Heart rate is continuously monitored using a 12-lead ECG. Respiratory data is continuously measured using breath-by-breath analysis (Oxycon Pro, Jaeger, CareFusion, Germany | JAEGER Vyntus CPX, Vyaire medical, United States). VO_2_peak is defined as the highest 30-s average of VO_2_ at the end of the test.

### Blood and urine sampling

Blood will be drawn in a fasted state by venipuncture of the cubital fossa for both routine analysis as well as circulating biomarkers of systemic CV risk, after at least 1 day of rest after the last physical exercise but within 1 week. This will be performed at baseline, after 3 months, 6 months and 12 months of trial inclusion. Urine will also be collected in a fastened state. Routine urine analysis will be used for the determination of microalbuminuria. Routine blood analyses encompass:

• Hemogram with differential blood cell count.
• Biochemistry: high-sensitive c-reactive protein (hsCRP), glucose, HDL, LDL, triglicerides, HbA1c, kidney markers (creatinine, sodium, potassium).
• Biomarkers of endothelial function and premature vascular ageing comprise different cellular (circulating mature endothelial and endothelial progenitor cells; only assessed in Basel) and protein markers, but also transcriptomics, genomics and epigenetic markers.
• ELISA kits will be used according to the manufacturers’ instructions for the determination of interleukine-6 (Bender Med-Systems, Austria), tumor necrosis factor-α (Biosource, United States), 3-nitrotyrosine (oxidative stress, Cell Biolabs, CA, United States), and BDNF (Biosensis, United States). High-sensitive c-reactive protein (hs-CRP) is analysed by immunoturbidimetric latex CRP assay (Cobas 8000, Roche-Diagnostics, Basel).

Blood samples are centrifuged, and the supernatant aliquots are frozen at −80°C for batch analysis.

#### Questionnaires

International Physical Activity Questionnaire (iPAQ): The short form of the iPAQ is used in the validated German and Dutch versions. It is applied to identify the quantity and kind of daily physical activity in all participants. From the questionnaire, the metabolic equivalents (MET) per 1 hour (met/h) can be calculated per week. The instrument has demonstrated good reliability and validity (Craig et al., 2003).

National Eye Institute 25-Item Visual Function Questionnaire (NEI VFQ-25): NEI VFQ-25 assesses vision related quality of life (vision comfort in daily activities as driving, newspaper reading and cinema) (Mangione et al., 2001).

Glaucoma Activity Limitation Questionnaire (GAL-9): GAL-9 is the shortened version of the Glaucoma Quality of Life-15 and also assesses vision related quality of life, specifically developed for glaucoma patients (Nayyar et al., 2022).

Ocular Surface Disease Index (OSDI): OSDI assesses the effects of dry eye disease on vision-related function (Guarnieri et al., 2020).

Five-item Dry Eye Questionnaire (DEQ-5): DEQ-5 will be used to discriminate self-assessed severity of dry eye disease (Chalmers et al., 2010).

#### Further assessments

Daily physical activity: physical activity at baseline, three and 6 months as well as 12 months of follow-up will be measured objectively through a validated research-based accelerometer (GENEActiv ActiGraph™ GT3X, ActiGraph LLC, Pensacola, Florida, United States), that will be worn for 8 consecutive days in order to obtain number of steps, energy expenditure, MET rates, physical activity intensity, sedentary bouts, activity counts and activity bouts.

24 h ambulatory blood pressure measurement: Patients will receive a validated oscillometric (either 90202 or 90207 SpaceLabs monitor (SpaceLabs Inc., Redmond, WA) or ABPMpro—Research model (SOMNOmedics GmbH, Randersacker, GER)) which will be programmed to obtain ambulatory blood pressure readings at intervals of 15 or 20 min during the day (from 06:00 to 00:00) and at 30-min intervals during the night (from 00:00 to 06:00).

Anthropometry: Patient’s height, weight, body mass index (BMI), resting electrocardiogram (ECG), and blood pressure (BP) will all be measured using standardized procedures. Body fat will be measured with bioelectrical impedance analysis (InBody, Biospace, California, United States).

### Data analysis

Primary outcome of the study is the effect of 6-month exercise training on maximal aFID and vFID in the HIIT group compared to the control group. Descriptive statistics (mean, standard deviation) will be used to describe baseline and follow-up characteristics. Normal distribution of data will be tested by means of Shapiro-Wilk test. To analyze intervention effects after 6 months, we will use an analysis of covariance (ANCOVA) according to the intention-to-treat principle to compare differences in primary and secondary endpoints between HIIT and control group, corrected for the corresponding baseline value and main confounders. Boxplots will be used to visualize primary and secondary endpoints before and after the 6-months exercise intervention. If the fraction of missing values exceeds 5%, we will account for missing data using multiple imputation. A multiple linear regression model, adjusted for covariables as age and sex, will be used to describe the association between the delta change of retinal microvascular adaptations and the delta change of classical risk factors such as VO_2_peak, BMI, systolic and diastolic BP, fasting glucose, serum lipids, hs-CRP, inflammatory and oxidative stress markers as well as BDNF. Changes in glaucoma therapy may coincide with the course of this project, as the trial does not interfere with routine glaucoma management. Therefore, there is a particular need to correct for therapy changes in the statistical analysis. Any changes in therapy will be included as separate variables. Any step-up of therapy, being either surgery, laser trabeculoplasty or additional drops, will be coded as a binary, summarizing variable (yes/no) and as separate variable (quantifying total number of drops, total number of surgeries). Model assumptions will be checked graphically using residual diagnostic plots. All statistical tests will be two‐sided and the significant level is set to 0.05.

### Sample size calculation

Retinal microcirculation: Our sample size calculations were based on the regression coefficient for the group indicator (HIIT vs. CG) in an ANCOVA as described above. Our calculations were informed by the published standard errors for these coefficients of comparable trials and analyses. Specifically, we assumed a standard error of 0.23 for maximal aFID (Streese et al., 2020a) and of 0.0051 for AVR (Streese et al., 2020b). To detect adjusted group differences of 0.51% for maximal aFID and 0.03 for AVR with a power of 80%, a total sample size of 112 and 20 is required, respectively. We therefore envisage at least 112 completed participations in the study.

In case we would not meet our sample size target, we also calculated the minimum detectable β for a range of sample sizes. In case of recruitment, for example, of only 90 patients, the minimum detectable β for maximal aFID would be about 0.60% exercise-induced dilation change for a power of 0.8, and the minimum detectable β for AVR would be in the range of 0.014 exercise-induced change in ratio. These would still reflect very achievable and clinically relevant improvements of retinal microvascular health, putting the expected effects into perspective of our normative data and previously reported exercise efficacy.

Large artery function: An additional sample size calculation was performed based on the given sample size from the previous power calculation for the retinal endpoint. Our detectable group difference calculations were based on an assumed correlation coefficient of r = 0.6 between pre- and post-measurement, a standard deviation of 3%, a given total sample size of n = 124 (62 per group; MCT/HIIT group) and a dropout rate of 10%. Our calculations were informed by the published standard deviation for these coefficients of comparable trials and analyses (Lenth, 2001; Moriguchi et al., 2005; Luk et al., 2012; Ras et al., 2013; Heiss et al., 2023). With n = 62 per group and an assumption of r = 0.6 between pre- and post-measurement, we have 80% power to detect group differences in FMD of about 1.3% or larger and 90% power to detect group differences in FMD of about 1.5% or larger. We therefore target a sample size of 124 participants for the study.

If the correlation between pre- and post-measurement does not reach r = 0.6, we could still detect group differences in FMD of 1.6% with 80% power in the worst case. This would still reflect very achievable and clinically meaningful improvements in large artery function, putting the expected effects into perspective of recently published reference ranges and serve as a target for further and more tailored cardiovascular risk stratification and prevention (Luk et al., 2012; Heiss et al., 2023).

#### Data management

Data management will be performed by experienced data scientists in compliance with the guidelines of good clinical practice in collaboration with the Clinical Trial Unit (CTU) of the University of Basel and the Clinical Trial Center (CTC) of the University Hospitals UZ Leuven. The same electronic case report form (eCRF) (RedCap) is used in both centers. For personal and sensitive data, we abide the General Data Protection Regulation 2016/67 and US FDA Good Clinical Practice guidelines. Different data sources will be collected. Patient data will be digitally recorded in the form of questionnaires, clinical examination reports and image files. The management includes consistency checks of data input. Personal data and findings of this survey will be digitally stored in an encrypted format and will be used exclusively for the study. Neither names nor initials, complete birth dates or other identifiers will be included in the encrypted code (pseudonomization). After data acquisition is completed, all statistical analyses will be performed solely with pseudonomized data sets. Pseudonomized data (numeric database) will be sent from Leuven to the coordinating center. Non-disclosure to third parties, as well as other restrictions, are stipulated in the informed consent form. After the study, the data will be filed in the archive of the DSBG and in KU Leuven servers dedicated to the Research Group of Ophthalmology. The Data Management Plan has been approved by the institutional research governance committees and ethics review authorities.

## Discussion

HIT-GLAUCOMA aims to establish exercise treatment as a viable new adjunct therapy modality for glaucoma. The results of the study have the potential to enforce a paradigm shift in the treatment of patients with glaucoma by additionally applying exercise treatment. Exercise treatment has the potential to improve microvascular endothelial function, improve ocular perfusion and induce anti-inflammatory as well as anti-oxidative capacities. Glaucoma could potentially be seen, at least in part, a sequel of systemic dysfunctional pathways and theoretical microvascular dysfunction. Exercise is a much underused and readily available treatment strategy with great potential as add on treatment on top of usual care.

This is, first and foremost, a clinical study elaborating on the potential exercise-induced mechanisms of treatment effects. Nonetheless, the study investigates local retinal as well as systemic pathophysiological and molecular mechanisms that may be involved in the exercise-induced amelioration of disease progression. In many ways, this is a comprehensive and highly innovative approach combining exercise and preventive medicine as well as retinal microvascular pathophysiology with clinical ophthalmology with the potential to set new standards and offer new options for the current treatment approaches in glaucoma.

**FIGURE 3:**
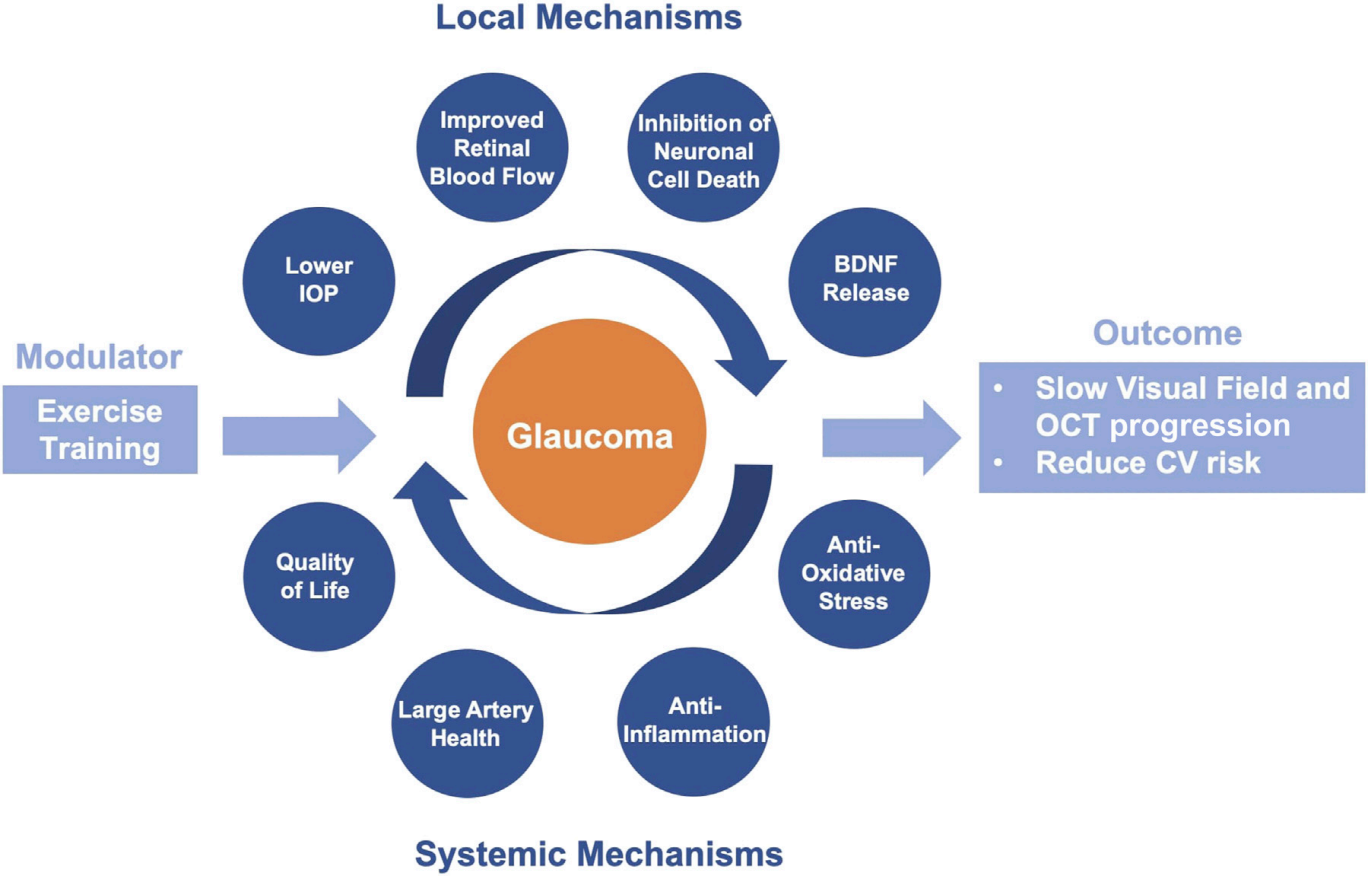
Visual representation of the study concept on the effects of exercise training on glaucoma progression and cardiovascular risk via local and systemic disease mechanisms.

The objectives of the HIT-GLAUCOMA study are primarily to investigate intermediate endpoints such as local retinal microvascular dysfunction and systemic vascular plasticity, inflammation, and oxidative stress. These are key pathological features within the etiology of glaucoma and describe local and systemic mechanisms that impact disease progression. We aim to demonstrate the efficacy of exercise as add-on treatment on these disease entities with the potential to help stabilize the disease and reduce glaucoma-associated CV risk. The aim and concept of the study is summarized in Figure 3. The study also investigates the effects of exercise on disease specific clinical endpoints such as visual field and OCT indices, IOP, use of IOP lowering eye drops or necessity for laser trabeculoplasty or surgical intervention as well as vision related quality of life and symptomatology of ocular surface disease. As the initial phase of the study is too short for robust and reliable study of glaucoma progression in terms of visual field and OCT, longer-term follow-up is ensured to assess these changes over a longer period. The first patients are scheduled in February 2024.

In conclusion, the comprehensive HIT-GLAUCOMA study approach will set the grounds for a new add-on exercise treatment regime for glaucoma and represents a blueprint of exercise study in all kinds of (micro)vascular diseases. Additionally, it will mobilize patients and benefit associated CV comorbidities. Exercise and its prescription could be considered an effective polypill for the treatment of systemic CV disease and may likewise prove to be an effective treatment strategy of glaucoma as a chronic smoldering neurodegenerative disease.

## Data Availability

The original contributions presented in the study are included in the article/Supplementary material, further inquiries can be directed to the corresponding authors.
